# Interaction and Reactivity of Cisplatin Physisorbed on Graphene Oxide Nano-Prototypes

**DOI:** 10.3390/nano10061074

**Published:** 2020-05-31

**Authors:** Ma del Refugio Cuevas-Flores, Massimiliano Bartolomei, Marco Antonio García-Revilla, Cecilia Coletti

**Affiliations:** 1 Departamento de Química, Universidad Autónoma de Zacatecas, 98000 Zacatecas, Mexico; qkis.cuevas@uaz.edu.mx; 2 Instituto de Física Fundamental, Consejo Superior de Investigaciones Científicas (IFF-CSIC), Serrano 123, 28006 Madrid, Spain; 3 Departamento de Química, DCNE, Universidad de Guanajuato, Noria Alta, 36050 Guanajuato, Mexico; magarcia@ugto.mx; 4 Dipartimento di Farmacia, Università degli Studi “G. d’Annunzio” Chieti-Pescara, 66100 Chieti, Italy; ccoletti@unich.it

**Keywords:** cisplatin, drug delivery, graphene, two-dimensional materials, ab initio calculations, intermolecular interactions

## Abstract

The physical adsorption of cisplatin (CP) on graphene oxide (GO) and reduced graphene oxide (rGO) is investigated at the DFT level of theory by exploiting suitable molecular prototypes representing the most probable adsorbing regions of GO and rGO nano-structures. The results show that the CP binding energy is enhanced with respect to that for the interaction with pristine graphene. This is due to the preferential adsorption of the drug in correspondence of the epoxy and hydroxy groups located on GO basal plane: an energy decomposition analysis of the corresponding binding energy reveals that the most attractive contribution comes from the electrostatic attraction between the -NH${}_{3}$ ends of CP and the oxygen groups on (r)GO, which can be associated with hydrogen bonding effects. Moreover, it is found that the reactivity of the physically adsorbed CP is practically unaltered being the free energy variation of the first hydrolysis reaction almost matching that of its free (unadsorbed drug) counterpart. The reported results suggest that the CP physical adsorption on GO and rGO carriers is overall feasible being an exergonic process in aqueous solution. The CP adsorption could facilitate its solubility and transport in water solutions, exploiting the high hydrophilicity of the peripheral carboxylic groups located on the edge of the GO and rGO nano-structures. Moreover, the the higher affinity of CP with respect to the oxidized sites suggests a possible dependence of drug loading and release on pH conditions, which would highly facilitate its specific delivery.

## 1. Introduction

Specific delivery is one of the main issues to be addressed to obtain the ideal pharmaceutical effect of drugs, that is to ensure that the desired biological target is preferentially reached, minimizing in this way possible collateral damages and undesired side effects. The latter [1] are particularly severe in the case of platinum-based chemotherapy, because of the high affinity of platinum(II) towards, especially, sulfur-containing aminoacids [2,3,4], with which it can react before reaching the final therapeutical target, i.e., cancer cell DNA. Protein-binding thus results in high general toxicity of Pt-based drugs, and, moreover, in the need of administrating larger doses to achieve the desired effect.

For this reason, molecular nano-carriers have been proposed for drug transportation, with the aim of specifically release of the loaded chemotherapic to cancer tissues, thus reducing the dose and enhancing the pharmaceutical activity [5]. Furthermore, suitably chosen nanocarriers, depending on their lipo- or hydro-philicity, can allow solubilization in different media and facilitate the drug administration.

Since their introduction, graphene-based biomaterials have shown excellent physico-chemical properties and have been widely tested as promising nanocarriers [6,7,8,9]. Indeed, their dominant two-dimensional character offers a large surface area-to-volume ratio, ideal for drug loading. Furthermore, they can be easily decorated by different functional groups with the possibility to modulate their properties, allowing them to improve their biocompatibility and toxicity [10,11]. Graphene Oxide (GO) in particular can present hydrophilic (epoxy, hydroxy, carboxylic, etc.) groups allowing an efficient dispersion in water and making it a very good candidate for drug transportation.

Though the exploration of Graphene-based structures as nanocarriers for cancer therapy is a relatively new field, Graphene and Graphene Oxides have been tested with extremely promising results for a number of molecules [12], including Cisplatin (CP) [13,14,15,16], i.e., cis-diamminedichloroplatinum(II), cis-[PtCl${}_{2}$(NH${}_{3}$)${}_{2}$], the first platinum-based molecule in clinical use and still one of the first choices, in combination with different drugs, in the treatment, among others, of ovarian, testicular and lung cancer [17,18]. The effectiveness of such combined formulation was observed by using graphene quantum dots [7], which showed an enhancement of the anticancer activity due to increased uptake of CP by tumour cells.

Most of the above-mentioned studies exploit the covalent binding of CP to the carbon surface. An alternative to the covalent binding for drug loading could involve its physisorption and recently we have defined a reliable methodology for the characterization and determination of noncovalent interactions between CP and Graphene finite prototypes [19], the latter acting as adsorbing platforms. The reported study shows an important affinity of CP for the adsorption on large area aromatic regions, as demonstrated by an estimated enthalpy variation of about −0.9 eV for the drug physisorption on Graphene.

It can be expected that even more favorable values would be found in the case of the CP adsorption on Graphene Oxide (GO) supports since hydrogen bond contributions arising from the presence of epoxy and hydroxy functional groups add up to ubiquitous Van der Waals forces. As a matter of a fact, Yang and co-workers [20] reported an experimental study in which hydrogen bond interactions can significantly contribute to the adsorption of doxorubicin on GO as shown from the dependence of the drug loading and release on pH conditions. More recently, a study highlighted that GO nanoplatelets are able to act as nanocarriers for CP and enhance its toxic effect on human lung cancer cells (A549 cells) and might similarly be dependent on the environmental pH [21]. GO-coated chitosan nanocomposite were also shown [22] to possess high cisplatin loading/releasing capacities likely connected to the favorable adsorption thermodynamics arising from hydrogen bond interactions.

The aim of this paper is, therefore, to theoretically assess the possibility of using GO and reduced GO (rGO) nano-prototypes for the efficient loading and release of the CP drug. Furthermore, we examine in detail the contributions of different kinds of noncovalent interactions on the overall binding. A deeper knowledge of the factors governing the binding mechanism can effectively suggest which functional groups and/or which contributions are more appropriate to modulate the GO nanocarriers behavior for a more specific and efficient drug loading/release process.

For the description of the structure of the GO prototypes, we have considered different models: the most popular is that of Lerf–Klinowski [23] which depicts it as a random distribution of epoxy and hydroxy groups on the basal plane of the carbon sheet. In this model, other oxidized groups (mostly the highly hydrophilic carboxylic acid group) are also present but in small quantities and located at the edges of the GO platelets. A more recent view [24] of the GO structure also considers that both large unoxidized (graphene-like) and oxidized (highly functionalized) regions can coexist. According to these models and for the sake of simplicity, we have taken into account three different sites on which the physisorption of CP is most probable: (i) a flat aromatic region (formed by sp${}^{2}$-hybridized carbon atoms); (ii) a flat carbon region with one epoxy group; (iii) a flat carbon region with one hydroxy group.

**Figure 1:**
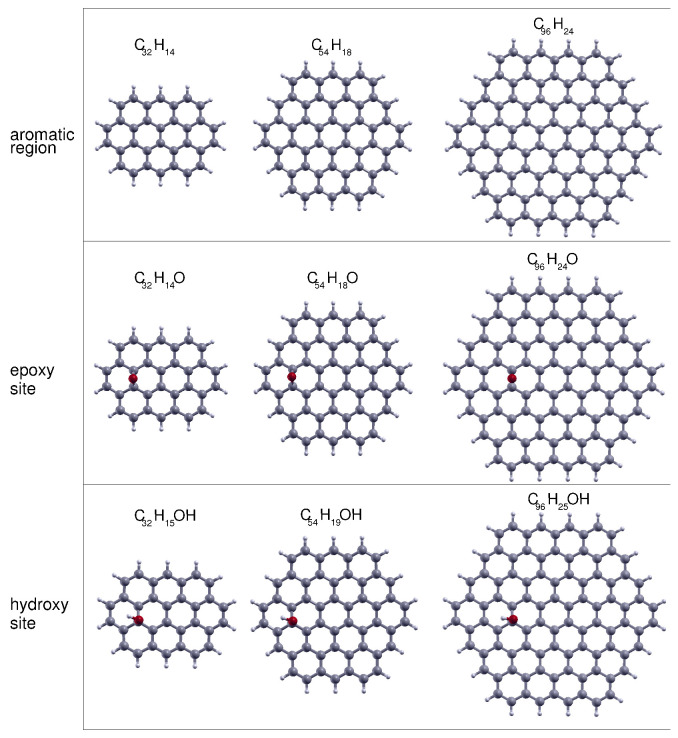
Graphene oxide molecular prototypes of increasing size. Those describing the aromatic region (first row) are ovalene (C${}_{32}$H${}_{14}$), circumcoronene (C${}_{54}$H${}_{18}$) and circumcircumcoronene (C${}_{96}$H${}_{24}$). The prototypes describing the oxidized sites are obtained by adding one epoxy (second row) or one hydroxy (third row) group to those of the first row.

The molecular prototypes chosen to represent these adsorbing regions are reported in Figure 1: a planar polycyclic aromatic hydrocarbon (PAH) of increasing size, from ovalene (C${}_{32}$H${}_{14}$) to circumcircumcoronene (C${}_{96}$H${}_{24}$) (first row), and their derivatives obtained by adding one epoxy (second row) and one hydroxy (third row) group oriented in a perpendicular fashion with respect to the carbon plane and laterally displaced from the center of mass, so to maximize the interaction of the adsorbate with both the functional group and the aromatic region of the carbon plane. This is particularly evident for the smallest prototypes based on ovalene for which the best choice is to place the oxidized groups in a mid-point between the edge and the center of the carbon plane along the largest molecular axis. For the remaining prototypes we have maintained the same decentred position of the functional groups to better study the convergence of the CP binding energy with the size of the carbon support.

The largest prototypes are also meant to better represent the structure of rGO, for which the carbon-to-oxygen ratio is quite high and correspondingly very few oxidized groups exist on the basal plane.

The paper is organized as follows. Section 2 refers the details of computational methodologies used for the electronic structure calculations. In Section 3 we present the results concerning both CP adsorption and reactivity. The paper ends with Section 4, in which some conclusions are summarized.

## 2. Computational Methods

The geometry optimization of the complexes formed by the CP adsorbed on the different GO prototypes has been performed at the density functional theory DFT level by considering the PBE [25] functional together with the latest dispersion contribution correction (D3 (BJ)) of Grimme [26], which includes the damped dispersion scheme of Becke–Johnson (BJ) [27]. In these DFT computations, the Pt atom has been described by means of the Stuttgart–Dresden pseudopotential [28] whereas for the rest of the atoms the 6–311+G [29] basis set (denoted as Ia) has been used: the geometry optimization together with the corresponding frequency calculations have provided the minimum (or transition state) structure and its energy. Intrinsic reaction coordinate (IRC) calculations have also been employed in order to correctly locate the reactives and product minima connected to the predicted transition state. The procedure used in this work was previously validated for the adsorption of a CP molecule on finite graphene prototypes [19].

In order to provide an estimation of the thermodynamic properties of the cluster in the gas phase at 298 K and 1 atm we have followed the guidelines reported in Refs. [19,30]: rigid rotor and harmonic oscillator approximations are assumed and frequency calculations have been performed by freezing the GO support (except for the epoxy or hydroxy group) while allowing the internal coordinates of the adsorbed species to relax. Once the stationary point has been found, the corresponding energy has been evaluated with a larger 6–311+G(2d,2p) [29] basis set (denoted as Ib) for the atoms others than Pt by performing single point calculations: CP adsorption and reaction enthalpy (free energy) have been determined by adding the corresponding zero-point energy and thermal (and entropy) corrections determined with the Ia basis set to the electronic energy variation obtained with the Ib one.

Additional computations of the interaction energies corresponding to the most stable configurations obtained at the PBE-D3 (BJ) level for the CP adsorption on the smallest prototype have also been carried out by using the B3LYP [31] (coupled with the D3 (BJ) dispersion correction) and M06-2X [32] DFT approaches together with the Ib basis set. Moreover, for the same configurations benchmark interaction energies have been computed at the “coupled” second-order Møller-Plesset perturbation theory (MP2C) [33] level of theory, which is capable to provide accurate results [34] for weakly bound systems [19,35,36,37,38,39] of different nature at an affordable computational cost: in particular, a complete basis set (CBS) estimation of the MP2C interaction energies has been obtained by exploiting the two-point correlation energy extrapolation of Halkier et al. [40,41] in conjunction with the Dunning aug-cc-pVDZ/aug-cc-pVDZ-PP and aug-cc-pVTZ/aug-cc-pVTZ-PP basis sets [42,43], as previosuly done in Ref. [19]. All reported interaction energies are defined as the energy difference between the complex and infinitely separated monomers having the same geometry than in the aggregate.

DFT and MP2C interaction energies have been corrected for the basis set superposition error (BSSE) by the counterpoise method of Boys and Bernardi [44].

In order to provide an estimation of the enthalpy (ΔH${}^{sol}$) and free energy (ΔG${}^{sol}$) variation in an aqueous solution we have adopted the following procedure. First, we have reoptimized the structures corresponding to the stationary points found in the gas phase calculations by using the SMD [45] continuum solvation model. This first step accounts for the hydration contribution to the enthalpy of the involved monomers, except for H${}_{2}$O and Cl${}^{-}$ for which the corresponding experimental values [46] are instead used.

Then, the Werz scheme [47] has been adopted to obtain a more reliable estimation of the entropy balance for the processes occurring in a water solution. The entropy variation so determined is therefore used to carry out the related free energy.

In order to make both DFT and MP2C calculations tractable, the density-fitting method [48] has been applied to approximate the two-electron repulsion integrals. MP2C and DFT computations have been performed by using the Molpro2012.1 [49] and Gaussian 09 [50] codes, respectively.

As a further matter, in order to get a deeper insight into the partial contributions to the total interaction energy, we have applied the Energy Decomposition Analysis (EDA) method [51,52], which is based on DFT and it is capable to provide reliable results related to the nature of the bonding in weakly interacting intermolecular complexes in the gas phase.

The EDA scheme, as it has been implemented in the ADF code [53,54,55], exploits the framework of the molecular orbital methods and it divides the total interaction energy between the GO prototypes and CP into three well-defined terms that can be interpreted in chemically meaningful ways. These terms are the electrostatic interaction energy between the charge densities of the monomers, ΔE${}_{elst}$, the energy gain due to orbital mixing of the monomers, ΔE${}_{orb}$, and the exchange repulsion between the monomers due to Pauli’s principle, ΔE${}_{pauli}$. An additional term, ΔE${}_{disp}$, that accounts for long-range dispersion effects, has been also included.

The EDA calculations have been carried out at the PBE-D3 (BJ) level of theory by exploiting the ADF code [53,54,55]: the Pt atom has been described by means of the ZORA pseudopotential [56] while for the rest of the atoms the Dunning aug-TZ2P basis set has been used.

## 3. Results and Discussion

### 3.1. Cisplatin Adsorption

Recently, we have found [19] that ovalene is a very suitable PAH in terms of binding energy convergence and related computational cost in order to study the noncovalent interactions between CP and a graphic plane and that the PBE-D3 (BJ) level of theory is capable of providing reliable estimations of the related binding energies.

**Figure 2:**
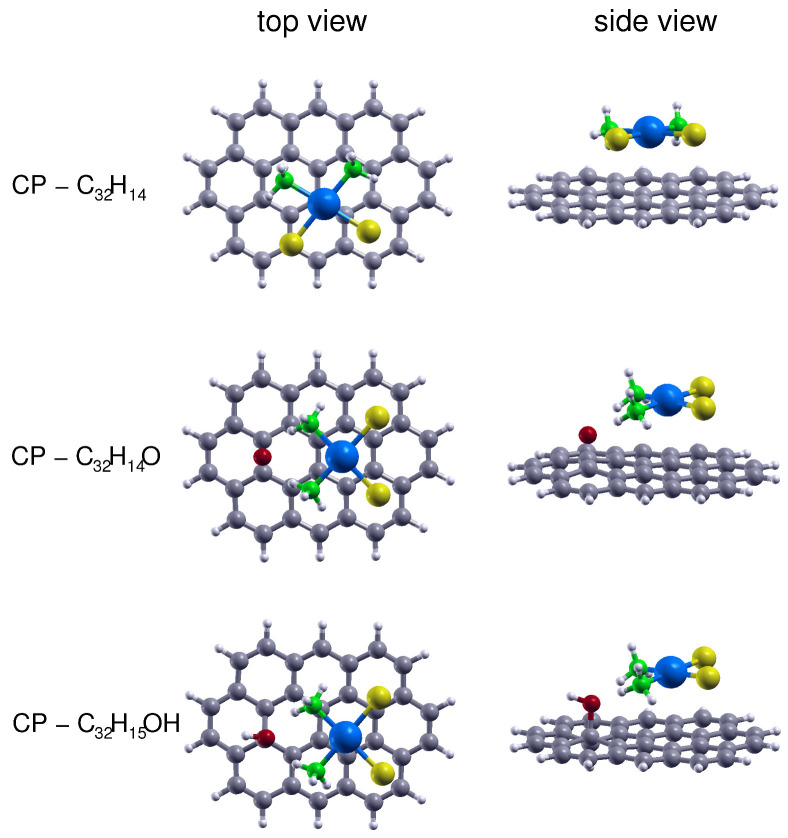
Top and side views of the CP–C${}_{32}$H${}_{14}$, CP–C${}_{32}$H${}_{14}$O and CP–C${}_{32}$H${}_{15}$OH complexes whose structures have been optimized by means of DFT computations at the PBE-D3 (BJ) level. The corresponding interaction energies are reported in Table 1.

The first step in the present work has been to validate the most adequate DFT level for the adsorption of CP on the GO prototypes containing the epoxy and hydroxy groups. To do that we have performed DFT optimizations at the PBE-D3 (BJ) level of the CP–C${}_{32}$H${}_{14}$O and CP–C${}_{32}$H${}_{15}$OH complexes whose most stable structures are reported in Figure 2 together with that previously obtained [19] for the CP–C${}_{32}$H${}_{14}$ cluster. The corresponding interaction energies are reported in Table 1 and they have been computed also at the B3LYP-D3 (BJ) and M062X levels, which are generally considered [34,57] as recommended DFT approaches to study noncovalently bonded intermolecular systems. From the comparison with the MP2C/CBS results, which can be considered here as the reference ones, it can be seen that for all cases the PBE-D3 (BJ) and M062X approaches globally provide an underestimation of the interaction energy, while the opposite is observed for the B3LYP-D3 (BJ) predictions, which lead indeed to larger discrepancies of about 150 meV. More in details, the PBE-D3 (BJ) results provide a better agreement (within 25 meV) for the interaction with the aromatic region GO prototype, which is consistent with our previous findings reported in Ref. [19], while those carried out at the M062X level better describe (within 20–25 meV) that with the other GO supports. In any case, the differences found for the PBE-D3 (BJ) estimations are globally below 50 meV (and around 4%) for all prototypes and, considering its lower computational cost over the M062X approach, we have decided to use it to perform the geoemetry optimization for the complexes involving larger GO prototypes, both in the gas phase and including an implicit solvent model. Moreover, this choice is motivated by the fact that the PBE-D3 (BJ) functional is capable to predict accurate thermodynamic properties for the cisplatin first hydrolysis reaction, as will be discussed in the next section.

As a result, in Table 2 we, therefore, present the interaction energy obtained for the adsorption of CP on GO molecular prototypes of increasing size. For circumcoronene and its epoxy(hydroxy) derivatives the reported energies correspond to the full optimization of the complex geometries. However, mostly motivated by the need to make the DFT computations affordable, in the case of circumcircumcoronene-based supports we have just retained the same intermolecular configurations found for the smaller (circumcoronene-based) GO platforms, considering also that significant changes in the CP adsorption geometry should not be expected. As a matter of fact, the relative geometries of the CP–C${}_{54}$H${}_{18}$ and CP–C${}_{54}$H${}_{18}$O(C${}_{54}$H${}_{19}$OH) complexes are very similar to those reported in Figure 2 and are not reported here for the sake of simplicity. CP–C${}_{54}$H${}_{18}$O and CP–C${}_{54}$H${}_{19}$OH geometries can be found in Figure S2 of the Supporting Information.

It can be seen that the interaction energy increases as the size of the GO prototypes become larger: in the case of functionalized sites, a more attractive binding of about 9 and 7% for the epoxy and hydroxy supports, respectively, is observed when going from C${}_{32}$H${}_{14}$ to C${}_{54}$H${}_{18}$, while for the largest prototypes (last row of Table 2) the corresponding improvement is of just about 2.4 and 1%. As for the aromatic region substrate, the gain in the interaction energy is similar and additional contributions of about 7 and 3 % are found.

These results suggest that the binding energy is well converged for the circumcircumcoronene-based adsorbing platforms and that it is likely that larger prototypes would lead to a slightly larger interaction of less than 1%, as already shown in Ref. [19] (see Table 2 therein) for graphene prototypes of increasing size.

In order to further assess the reliability of the considered GO molecular models we have considered four additional prototypes based on C${}_{54}$H${}_{18}$: Two of them are characterized by the functional group attached on the most central carbon ring; the other two also consider two different oxygen groups attached to the carbon basal plane and close to each other.

The optimized structures of the complexes between CP and these additional prototypes, which are also meant to take into account adsorption sites on GO with a high density of oxidized groups, are shown in Figure S1 of the Supporting Information. The corresponding total interaction energies are reported in Table S1 of the Supporting Information and they are close to those reported in the second row of Table 2 and related to the mono-(and “decentred”-)functionalized prototypes based on C${}_{54}$H${}_{18}$. These findings demonstrate that the physisorption of cisplatin does not significantly depend on the location of the oxidized group (as long as it is far from the plane edges). Moreover, they also show that the drug interaction with two contiguous oxygen groups leads to a negligible improvement of its adsorption energy with respect to that arising with a single oxygen group.

A careful look at Figure 2 suggests that the nature of the binding for the CP adsorption on the considered GO prototypes must be different: even if in all cases the -NH${}_{3}$ ends of CP lean toward the carbon plane, with the Pt atom separated from it of about 3.4 Å, in the case of the the aromatic region the hydrogen atoms tend to interact with the π cloud, whereas for functionalized sites they are oriented in the direction of the epoxy and hydroxy oxygens.

In order to get insight into the nature of binding, an analysis of the energy contributions to the chemical interaction has been performed using the EDA approach [51,52], which is an alternative [58] to the canonical DFT-SAPT [59] method, when the use of the latter is not computationally convenient. The agreement between corresponding energy contributions as predicted by the EDA and SAPT-based methods are in general qualitatively good since both approaches characterize the noncovalent character of the chemical binding in a very similar way [58]. In order to validate this assumption, an EDA analysis has been first carried out for the smaller CP-Pyrene (CP-P) complex and the results have been compared with those previously reported at DFT-SAPT level [19], finding that the partial contributions are qualitatively similar around equilibrium distances, with the dispersion plus induction (or orbital) energies being the main contribution to the total interaction energy (see Table S2 in the Supporting Information for more details).

These results allowed us to safely apply the EDA approach to the total interaction energy found for CP adsorbed on larger prototypes. In particular, in Table 3 we report the partial contributions related to the optimized geometries of the CP–C${}_{54}$H${}_{18}$ complexes, which can be considered sufficiently large and representative of the overall interaction acting between CP and GO. Significant differences can be indeed seen between the aromatic and the oxidized prototypes: the total interaction energy of CP adsorbed on the aromatic prototype is about −900 meV while for that for the epoxy and hydroxy functionalized supports become larger of about 50% in both cases.

These differences can be related to the specific role of the different energy contributions. More in details, it can be appreciated that the main attractive contribution to the total interaction energy has changed in the case of the functionalized prototypes: while the dispersion energy term remains almost unperturbed, the electrostatic contribution becomes dominant since it displays an increase of 119% and 155% for the epoxy and hydroxy supports, respectively. Regarding the Pauli repulsive contribution, it rises of about 50% and 73% for epoxy and hydroxy prototypes, respectively, but this increase is mostly compensated in both cases by the enlargment of the orbital attractive term.

Therefore, while the dominant attractive contribution for the CP-aromatic prototype complex is the dispersion interaction, which only shows a marginal increase in epoxy and hydroxy species, for the oxidized prototypes the electrostatic contribution is the most important one and sums up leading to significantly larger total binding energies. This enhanced electrostatic contribution can be related to the hydrogen bond-like interaction arising between the -NH${}_{3}$ ends of CP and the oxygen groups on the carbon supports. In order to confirm such hypothesis calculations on intermolecular prototypes, such as NH${}_{3}$-H${}_{2}$O, NH${}_{3}$-OHCH${}_{3}$ and NH${}_{3}$-O( CH${}_{3}$)${}_{2}$ (see also Figure S2 in the Supporting Information), representing archetypal hydrogen bonds have been carried out. Similar EDA results as for the CP-oxidized prototypes interaction have been obtained and the greatest contribution to the total interaction energy corresponds to the electrostatic term (see Table S3 in the Supporting Information). In addition, for these archetypal systems, the O–H distance (between the hydrogen of the ammonia and oxygen of the hydroxy and epoxy groups) is around 2 Å while the N–H–O angle is about 160°. Such values are very similar to those obtained for the CP-GO prototypes complexes for which we find distances of about 2.0 and 1.95 Å and corresponding angles of about 161° and 159° for the epoxy and hydroxy prototypes, respectively (see Figure S2 in the Supporting Information). Therefore, it can be concluded that the enhancement of CP physisorption on GO oxidized prototypes is mainly due to the formation of hydrogen bonds in which hydrogens of the CP ammonia groups interact with the oxygen of the hydroxy and epoxy groups.

**Figure 3:**
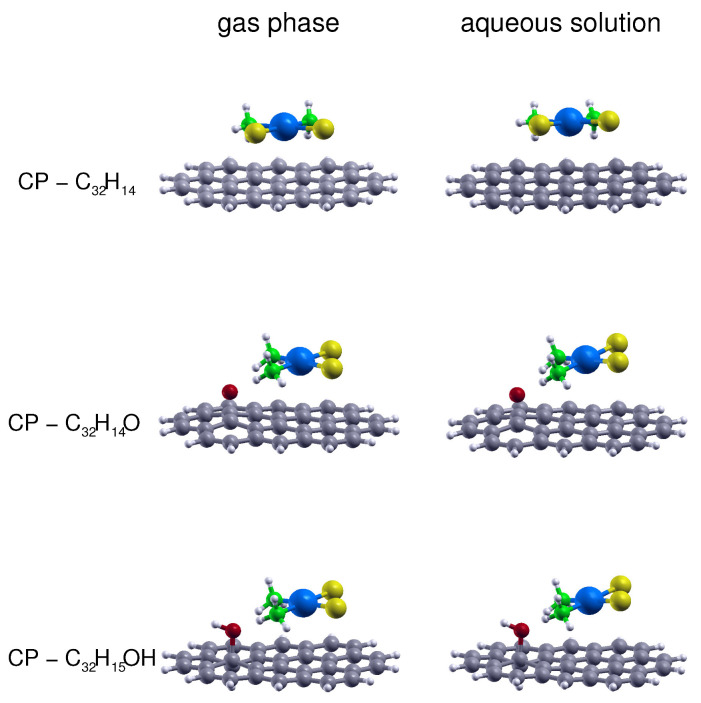
Side views of the CP–C${}_{32}$H${}_{14}$, CP–C${}_{32}$H${}_{14}$O and CP–C${}_{32}$H${}_{15}$OH gas phase optimized structures together with those obtained in aqueous solution by considering the SMD continuum solvation model.

To better assess the viability of the CP adsorption in a more realistic medium such as an aqueous solution we have performed geometry optimizations by means of the SMD continuum solvation model, whose results are shown in Figure 3 together with the corresponding gas-phase estimations: it can be appreciated that both models provide quite similar configurations although a small increase in the angle between the CP and the GO prototype can be appreciated for the SMD results, which leads to a slightly larger distance (of about 0.15 Å) between the Pt and the aromatic C plane. This suggests that the nature of the bonding should not significantly change in the gas phase and water solution. In Table 4 we finally report present estimations for the adsorption enthalpy (ΔH${}_{ads}$) and free energy (ΔG${}_{ads}$), both in gas phase and aqueous solution, computed at 298.15 K and 1 bar for CP physically sorbed on GO and on rGO. For the adsorption on GO we have chosen the results obtained for the circumcoronene-based supports. The predicted values for rGO have been obtained from the circumcoronene results by scaling the adsorption enthalpies using the interaction energies computed for the related C${}_{54}$H${}_{18}$ and C${}_{96}$H${}_{24}$ supports, which are reported in Table 2. It can be seen that for all considered sites the CP adsorption on GO and rGO is a significantly exothermic and exoergonic process in both the gas phase and aqueous solution. As expected, the most favorable adsorption occurs in correspondence of the hydroxy group, followed by the expoxy and aromatic region sites. In particular, ΔH${}_{ads}$ and ΔG${}_{ads}$ values related to the aqueous solution CP adsorption in correspondence of the oxidized sites are significantly larger (between 10 and 30% and between 15 and 45%, respectively) than those for the aromatic region, confirming the fundamental role of hydrogen bonding contributions also in the condensed phase. An indication of such favorable enthalpy and free energy adsorption variations, though calculated at a much lower and only qualitative level of theory (B3LYP/3-21G (d)), also emerged in the case of GO-coated chitosan, due to hydrogen bonding between the cisplatin ammonia and GO epoxy and hydroxy groups [22].

In order to take into account the possibility of the competition of the CP self-clusterization process, enthalpy and free energy values obtained for the CP dimerization are also reported in Table 4. It can be seen that in water solution the free energy variation for CP dimerization is lower or comparable with respect to those corresponding to the CP adsorption on the hydroxy and epoxy sites, whereas it is slightly larger (around 40–60 meV) than that for the aromatic region case.

In conclusion, the above reported results suggest that, on the whole, the CP physical adsorption on GO and rGO is competitive with the drug self-aggregation and it can help to increase its solubility in water solutions, exploiting the high hydrophilicity of the peripheral carboxylic groups located on the edge of the GO and rGO platelets. Moreover, the higher affinity of CP adsorption in correspondence of the oxidized sites, due to active hydrogen bonding contributions, suggests the possible dependence of drug loading and release on pH. This would be a particularly valuable feature, because it can highly enhance the specificity of CP loaded nanocarriers for tumor cells. The hypoxic, acidic environment of cancerous tissue should indeed be able to induce drug release by substituting the weaker hydrogen bonds between -NH${}_{3}$ groups and oxidized functions of (r)GO with stronger hydrogen bonds involving more acidic protonated groups. At physiological pH, on the other hand, CP molecules would remain adsorbed onto the surface, decreasing the possibility of undesired collateral reactions. Such a pH-dependent behavior was in fact found [20] in the case of doxorubicin noncovalently bound to GO supports, which show maximum loading capacity in basic solutions and minimum in acidic environments.

### 3.2. Cisplatin Reactivity

In order to address the reactivity of the physically adsorbed CP we have studied its first hydrolysis reaction which is generally considered [60] as the limiting step requiring the largest activation energy and leading to the monoaqua complex. The latter is, together with the diaqua analog, the reactive species responsible of the Pt covalent binding to DNA nucleobases and eventually of CP cytotoxicity.

**Figure 4:**
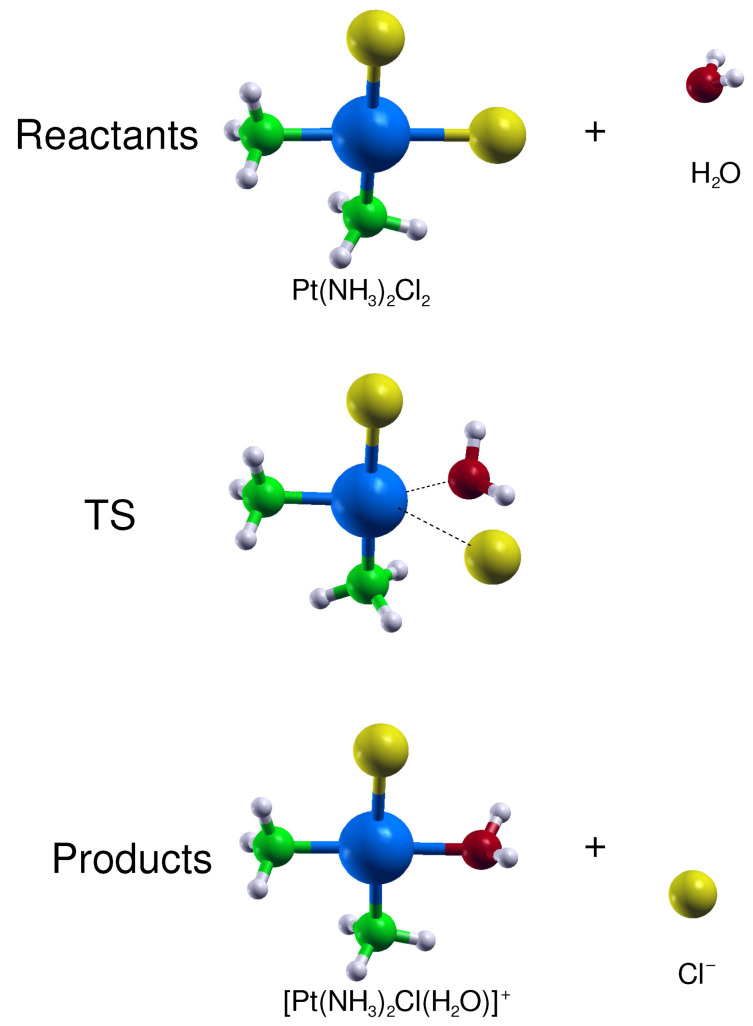
Side views of the reactants, transition state (TS) and products optimized structures related to the CP hydrolysis reaction. The reported structures are those obtained in aqueous solution by considering the SMD continuum solvation model.

First, we have tested the capacity of the present DFT approach (adopted in the previous section to study the cisplatin physical adsorption) to predict the drug activation and reaction energies. Cisplatin first hydrolysis proceeds through a S${}_{N}$2 substitution in which the nucleophile water molecule displaces the chloride ion leaving group (see Figure 4) and involves a distorted trigonal-bipyramidal transition state (TS) [61,62,63].

The enthalpy and free energy variations corresponding to the hydrolysis reaction and its activation for a free (unadsorbed) CP molecule are reported in Table 5. It can be seen that the estimations obtained with the present PBE-D3 (BJ)/SMD model are close to the experimental findings in aqueous solution and they can be considered of comparable or even better accuracy with respect to previous theoretical predictions [62,64]: in fact, present predictions provide a better accord for the ΔH${}_{r}^{sol}$ and ΔG${}_{r}^{sol}$ values since they are able to reproduce both the slight endothermicity and endergonicity of the hydrolysis reaction.

**Figure 5:**
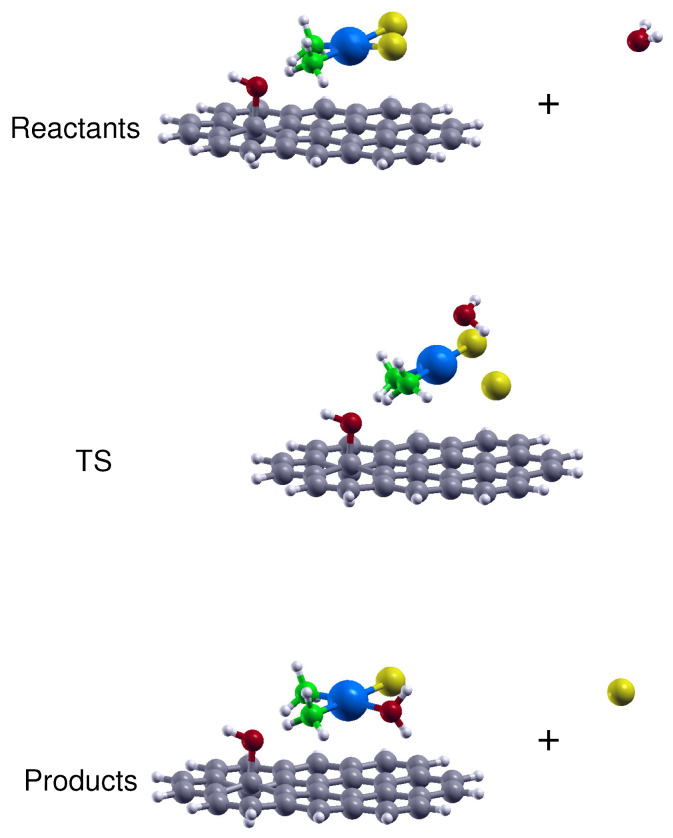
Side views of the reactants, transition state (TS) and products optimized structures related to the CP hydrolysis reaction with the drug adsorbed on a hydroxy site of a GO (or rGO) platelet. The reported structures are those obtained in aqueous solution by considering the SMD continuum solvation model.

Therefore, we believe that the present DFT approach is adequate to study the CP hydrolysis thermochemistry and we have also employed it to investigate the drug reactivity when it is physically adsorbed on GO and rGO platelets. To this aim, in order to make the optimization of the TS and products structures computationally affordable we have just considered the adsorption on the prototypes based on C${}_{32}$H${}_{14}$ (see the first column of Figure 1). The structures of the stationary points corresponding to the CP hydrolysis reactants, transition state and products, when adsorbed on the hydroxy site of a GO (or rGO) platelet are shown in Figure 5 while the related enthalpy and free energy variations in aqueous solution are reported in Table 6 together with those for the aromatic region and epoxy site.

It can be appreciated that negligible changes in the activation enthalpy and free energy are found with respect to the case of the free (unadsorbed) CP. In the case of the global reaction a slight lower endothermic and endergonic (of about 100 meV) process is found for the adsorbed drug. Therefore present findings indicate that the hydrolysis of CP is little affected by the condition of its physical adsorption on GO and rGO platelets. However, it must be pointed out that an effect of the reaction kinetics should be expected due to steric assumptions: in fact, when the CP is adsorbed, one of the two sides of the planar molecule is not available for the nucleophile approach which should, therefore, affect the reaction rate.

## 4. Conclusions

We have shown by means of DFT computations that CP can be efficiently physisorbed on GO and rGO nano-structures and that the related binding energy is enhanced with respect to the interaction with the pristine graphene surface. In particular, the most favorable adsorption occurs in correspondence of the epoxy and hydroxy groups located on the GO basal plane and an EDA interaction energy analysis reveals that, in those cases, the dominant attractive contribution comes from the electrostatic attraction between the -NH${}_{3}$ ends of CP and the oxygen groups on (r)GO. The increased role of this interaction component can be indeed associated to hydrogen bonding effects, as shown from the comparison with archetypal intermolecular systems. Globally, the CP physisorption on GO and rGO carriers is an exergonic process in the aqueous solution and it is competitive with the drug self-aggregation, which can help to increase its solubility and facilitate its transport, especially if GO nanoplatelets with specific base size [21] are conveniently chosen or if GO is properly functionalized with biocompatible polymers [68] such as poly(ethylene glycol). It is also demonstrated that the reactivity of the adsorbed CP is practically unaltered with respect to that of its free (unadsorbed drug) counterpart. Finally, the higher affinity of CP with respect to the oxidized sites on GO suggests a possible dependence of drug loading and release on pH conditions which could be exploited for its specific and efficient delivery. The methodology introduced here could be easily applied to investigate and predict the GO affinity for other metal-containing drugs. Work in this direction is in progress.

## Figures and Tables

**Table 1:** Total energy for the interaction of cisplatin (CP) adsorbed on the smallest graphene oxide (GO) molecular prototypes (C${}_{32}$H${}_{14}$, C${}_{32}$H${}_{14}$O and C${}_{32}$H${}_{15}$OH). The reported values correspond to the most stable configurations reported in Figure 2 and obtained at the PBE-D3 (BJ) level. Estimations at the B3LYP-D3 (BJ) and M062X DFT levels are also given as well as the reference MP2C value obtained considering a complete basis set (CBS) extrapolation. All values are in meV (1 kcal/mol = 43.37 meV) and are corrected to account for the basis set superposition error (BSSE).

		CP–C${}_{32}$H${}_{14}$	CP–C${}_{32}$H${}_{14}$O	CP–C${}_{32}$H${}_{15}$OH
PBE-D3 (BJ)		−744.7	−1125.9	−1230.8
B3LYP-D3 (BJ)		−916.4	−1333.6	−1431.2
M062X		−696.1	−1159.3	−1253.4
MP2C/CBS		−769.6	−1176.5	−1277.4

**Table 2:** Total energy for the interaction of CP adsorbed on GO molecular prototypes of increasing size deriving from ovalene (C${}_{32}$H${}_{14}$), circumcoronene (C${}_{54}$H${}_{18}$) and circumcircumcoronene (C${}_{96}$H${}_{24}$) (see Figure 1). In the case of ovalene and circumcoronene the interaction energy is that corresponding to the most stable complex structure optimized at the PBE-D3 (BJ) level; for circumcircumcoronene the same relative adsorbate-support configuration obtained for circumcoronene is retained. All values are in meV (1 kcal/mol = 43.37 meV) and BSSE corrected and uncorrected (in parentheses) interaction energies are given

		Aromatic Region		Epoxy Site		Hydroxy Site	
CP–C${}_{32}$H${}_{14}$		−744.7	(−866.5)	−1125.9	(−1261.2)	−1230.8	(−1370.9)
CP–C${}_{54}$H${}_{18}$		−794.1	(−939.4)	−1237.8	(−1389.1)	−1322.4	(−1475.9)
CP–C${}_{96}$H${}_{24}$		−818.8	(−963.7)	−1268.1	(−1422.5)	−1336.7	(−1492.4)

**Table 3:** Partition of the total energy for the interaction of CP adsorbed on circumcoronene-based prototypes (see Figure 1), as predicted by the EDA scheme. The energies were computed for the most stable complex structure. In parentheses the percent increase of the partial energy with respect to the aromatic region prediction is also shown. All values are in meV (1 kcal/mol = 43.37 meV) and are not corrected for the BSSE.

		Aromatic Region	Epoxy Site		Hydroxy Site	
ΔE${}_{elst}$		−470.0	−1029.8	(119%)	−1199.7	(155%)
ΔE${}_{pauli}$		841.2	1268.7	(51%)	1456.9	(73%)
ΔE${}_{orb}$		−607.0	−910.1	(50%)	−973.9	(60%)
ΔE${}_{disp}$		−686.8	−714.6	(4%)	−741.9	(8%)
ΔE${}_{total}$		−922.3	−1385.6	(50%)	−1458.6	(58%)

**Table 4:** Enthalpy (ΔH${}_{ads}$), and free energy (ΔG${}_{ads}$) variations for the physisorption of CP on GO supports and the related extrapolations for rGO. ΔH${}_{ads}$ and ΔG${}_{ads}$ refer to 298.15 K and 1 bar and have been obtained by assuming rigid rotor and harmonic frequency approximations through PBE-D3 (BJ) calculations by considering the carbon support as a rigid structure. Extrapolated values for the adsorption on rGO are obtained by scaling the adsorption enthalpies using the interaction energies computed for C${}_{54}$H${}_{18}$ and C${}_{96}$H${}_{24}$ (see text and Table 2). Corresponding values for the CP dimerization are also reported for comparison. The aqueous solution (sol) results refer to geometry optimizations carried out by using the SMD continuum solvation model. All values are in meV (1 kcal/mol = 43.37 meV).

	Gas Phase		Aqueous Solution	
	ΔH${}_{\mathrm{ads}}$	ΔG${}_{\mathrm{ads}}$	ΔH${}_{\mathrm{ads}}^{\mathrm{sol}}$	ΔG${}_{\mathrm{ads}}^{\mathrm{sol}}$
CP−GO(aromatic region)	−864.7	−396.6	−564.3	−308.2
CP−GO(epoxy site)	−1036.8	−532.4	−624.2	−348.5
CP−GO(hydroxy site)	−1192.0	−657.0	−748.3	−456.0
CP−rGO(aromatic region)	−889.0	−420.9	−588.6	−332.5
CP−rGO(epoxy site)	−1070.2	−565.8	−657.6	−381.9
CP−rGO(hydroxy site)	−1208.5	−673.5	−764.8	−472.5
CP−CP	−1870.5	−1278.9	−695.9	−373.1

**Table 5:** Activation enthalpies (ΔH${}_{\neq }$) and free energies (ΔG${}_{\neq }$) variations for the first hydrolysis reaction of CP in both gas phase and aqueous solution. Corresponding reaction enthalpies (ΔH${}_{r}$) and free energies (ΔG${}_{r}$) are also reported. Aqueous solutions predictions from Refs. [62,64] were obtained by using the Poisson–Boltzman (PB) continuum solvent model. All values are in eV (1 kcal/mol = 0.043 eV).

Gas Phase		ΔH${}_{\neq }$	ΔG${}_{\neq }$	ΔH${}_{r}$	ΔG${}_{r}$
	present	0.54	1.00	5.30	5.40
	B3LYP [62]	0.71	1.20	5.03	5.13
	B3LYP [64]		1.26		5.16
**aq. solution**		Δ **H** ${}_{\neq }^{\mathrm{sol}}$	Δ **G** ${}_{\neq }^{\mathrm{sol}}$	Δ **H** ${}_{r}^{\mathrm{sol}}$	Δ **G** ${}_{r}^{\mathrm{sol}}$
	present	0.78	1.03	0.20	0.26
	B3LYP/PB [62]	0.79	1.06	−0.04	0.01
	B3LYP/PB [64]		1.08		0.00
	Exp. [65,66,67]	0.82, 0.87, 0.95	1.01, 1.03, 1.05	0.13	0.16, 0.18

**Table 6:** Aqueous solution activation enthalpies (ΔH${}_{\neq }^{sol}$) and free energies (ΔG${}_{\neq }^{sol}$) variations for the first hydrolysis reaction of CP physically adsorbed on GO and rGO. Corresponding reaction enthalpies (ΔH${}_{r}^{sol}$) and free energies (ΔG${}_{r}^{sol}$) are also reported. The reported values refer to the adsorption on C${}_{32}$H${}_{14}$-based prototypes (see first column of Figure 1). All values are in eV (1 kcal/mol = 0.043 eV).

	ΔH${}_{\neq }^{\mathrm{sol}}$	ΔG${}_{\neq }^{\mathrm{sol}}$	ΔH${}_{r}^{\mathrm{sol}}$	ΔG${}_{r}^{\mathrm{sol}}$
free CP	0.78	1.03	0.20	0.26
adsorbed CP (aromatic region)	0.78	1.03	0.10	0.19
adsorbed CP (epoxy site)	0.79	1.05	0.13	0.22
adsorbed CP (hydroxy site))	0.77	1.02	0.10	0.18

## Supplementary Materials

The following are available online at https://www.mdpi.com/2079-4991/10/6/1074/s1, Additional figures and tables showing optimized structures, binding energies (and their partition in the main interaction contributions) for CP adsorbed on additional GO prototypes and for complexes involving ammonia and representing archetypal hydrogen bonds. Geometries of the optimized CP–C${}_{54}$H${}_{18}$, CP–C${}_{54}$H${}_{18}$O and CP–C${}_{54}$H${}_{19}$OH complexes.
