# Supplementary material for: Interaction and Reactivity of Cisplatin Physisorbed on Graphene Oxide Nano-Prototypes
Source: Nanomaterials (Basel). 2020 May 31;10(6):1074. doi: 10.3390/nano10061074 (PMC7353156; doi:10.3390/nano10061074)
Supplement: Supplementary file 1 [file nanomaterials-10-01074-s001.zip › nanomaterials-775748-suppl/nanomaterials-775748-suppl..pdf]

Supporting Information: Interaction and reactivity of cisplatin physisorbed on graphene oxide nano-prototypes

Ma del Refugio Cuevas-Flores

Departamento de Química, Universidad Autónoma de Zacatecas, Zacatecas, Mexico

Massimiliano Bartolomei *

Instituto de Física Fundamental, Consejo Superior de Investigaciones Científicas (IFF-CSIC), Serrano 123, 28006 Madrid, Spain

Marco Antonio Garcia Revilla

Departamento de Química, Universidad de Guanajuato, Guanajuato, Mexico

Cecilia Coletti

Dipartimento di Farmacia, Università degli Studi "G. D'Annunzio" Chieti-Pescara, 66100 Chieti, Italy

(Dated: May 3, 2020)

PACS numbers:

* Corresponding author, e-mail:maxbart@iff.csic.es

TABLE S1: Total energy for the interaction of CP adsorbed on further four different GO molecular prototypes (see Fig. S1) based on circumcoronene ($C_{54}H_{18}$). The first two ((a) and (b)) feature one functional group attached on the most central carbon ring. The last two ((c) and (d)) are characterized by one epoxy and one hydroxy group close to each other. All values are in meV (1 kcal/mol=43.37 meV) and BSSE corrected and uncorrected (in parentheses) interaction energies are given. These values should be compared with those reported in Table 2 (second row) of the manuscript.

(a) CP-$C_{54}H_{18}O$	-1253.0 (-1396.9)
(b) CP-$C_{54}H_{19}OH$	-1282.9 (-1428.2)
(c) CP-$C_{54}H_{19}OOH$	-1339.7 (-1498.0)
(d) CP-$C_{54}H_{19}OOH$	-1259.0 (-1406.9)

[1] M. Cuevas-Flores, M. Garcia-Revilla, and M. Bartolomei, J. Comput. Chem. **39**, 71 (2018).

TABLE S2: Comparison between DFT-SAPT contributions to the total interaction energies and related energy partitioning as predicted by the EDA scheme. The analysis refers to the parallel A2 and perpendicular B1 configurations of the CP-pyrene complex (see Fig. 1 of Ref.[1]). The DFT-SAPT values are those already reported in Table 1 of Ref.[1] and they correspond to intermolecular distances equals to 3.5 and 4.5 Å, roughly corresponding to the equilibrium distances for the A2 and B1 configurations, respectively. All values are in meV (1 kcal/mol=43.37 meV).

A2 (3.5 Å)				B1 (4.5 Å)			
DFT-SAPT		EDA		DFT-SAPT		EDA	
E_{Elst}	-279.9	ΔE_{elst}	-318.8	E_{Elst}	-388.0	ΔE_{elst}	-407.8
$E_{Exch-rep}$	672.8	ΔE_{pauli}	526.7	$E_{Exch-rep}$	611.9	ΔE_{pauli}	487.0
E_{Ind}	-214.9	ΔE_{orb}	-377.5	E_{Ind}	-170.6	ΔE_{orb}	-310.3
E_{Disp}	-750.0	ΔE_{disp}	-439.1	E_{Disp}	-557.3	ΔE_{disp}	-330.1
$E_{Ind+Disp}$	-964.9	$\Delta E_{orb+disp}$	-816.6	$E_{Ind+Disp}$	-727.9	$\Delta E_{orb+disp}$	-640.4
$E_{attraction}$	-1244.8	$\Delta E_{attraction}$	-1135.4	$E_{attraction}$	-1115.9	$\Delta E_{attraction}$	-1048.2
E_{total}	-571.7	ΔE_{total}	-608.7	E_{total}	-504.0	ΔE_{total}	-561.2

TABLE S3: Partition of the total energy for the interaction within intermolecular prototypes (see also Fig. S2) representing archetypal hydrogen bonds, as predicted by the EDA scheme. The energies were computed for the most stable complex structure. All values are in meV (1 kcal/mol=43.37 meV).

	NH_3-H_2O	NH_3-$OHCH_3$	NH_3-$O(CH_3)_2$
ΔE_{elst}	-161.3	-211.2	-308.7
ΔE_{pauli}	118.8	172.1	300.9
ΔE_{orb}	-58.9	-84.1	-146.6
ΔE_{disp}	-16.5	-32.9	-68.5
ΔE_{total}	-117.9	-155.7	-222.9

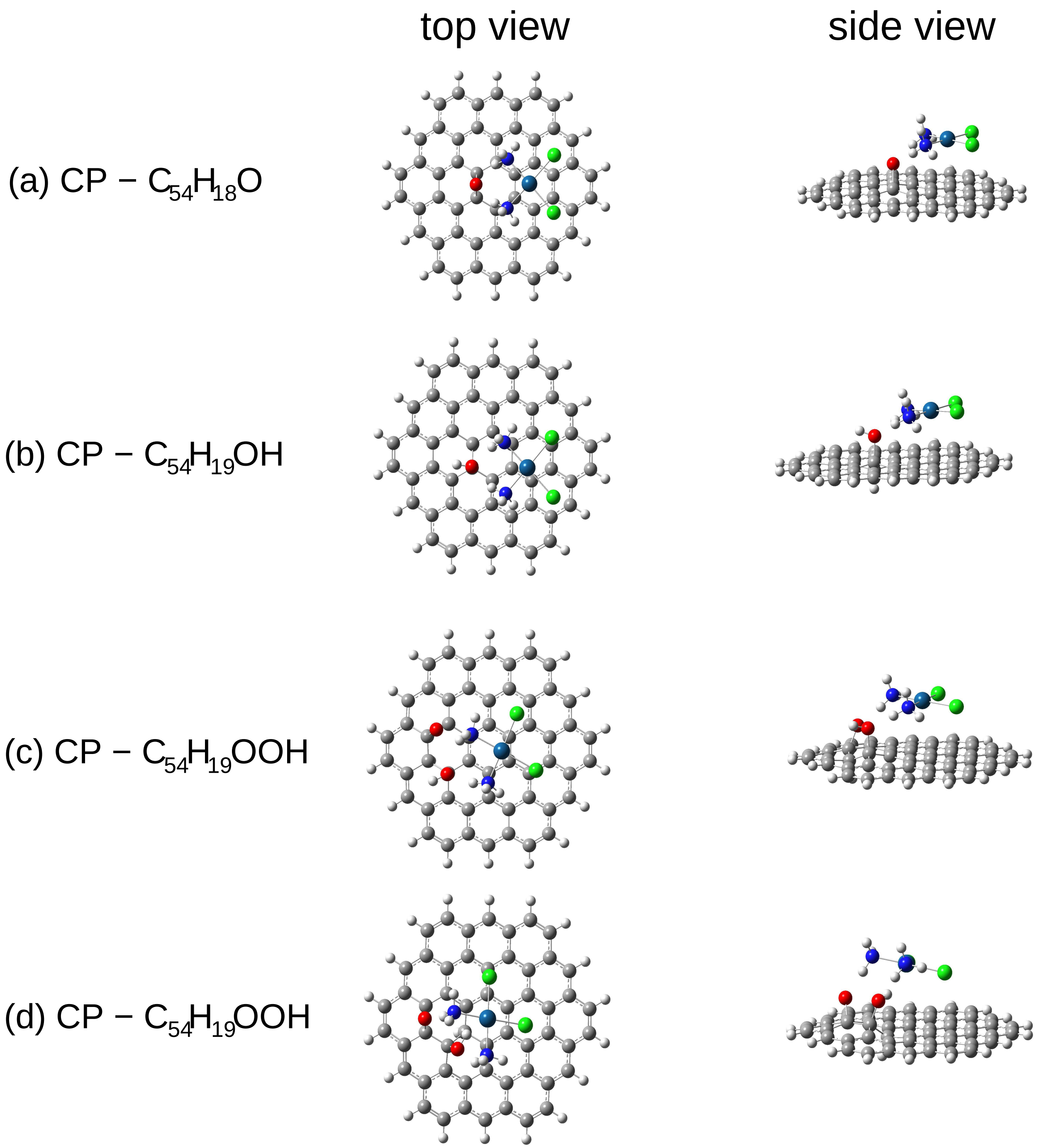

FIG. S1: Top and side views of four different complexes between CP and GO prototypes based on $C_{54}H_{18}$. (a) and (b) feature one functional group attached on the most central carbon ring. (c) and (d) are characterized by two different arrangements of one epoxy and one hydroxy group close to each other. The related structures have been optimized by means of DFT computations at the PBE-D3(BJ) level. The corresponding interaction energies are reported in Table S1.

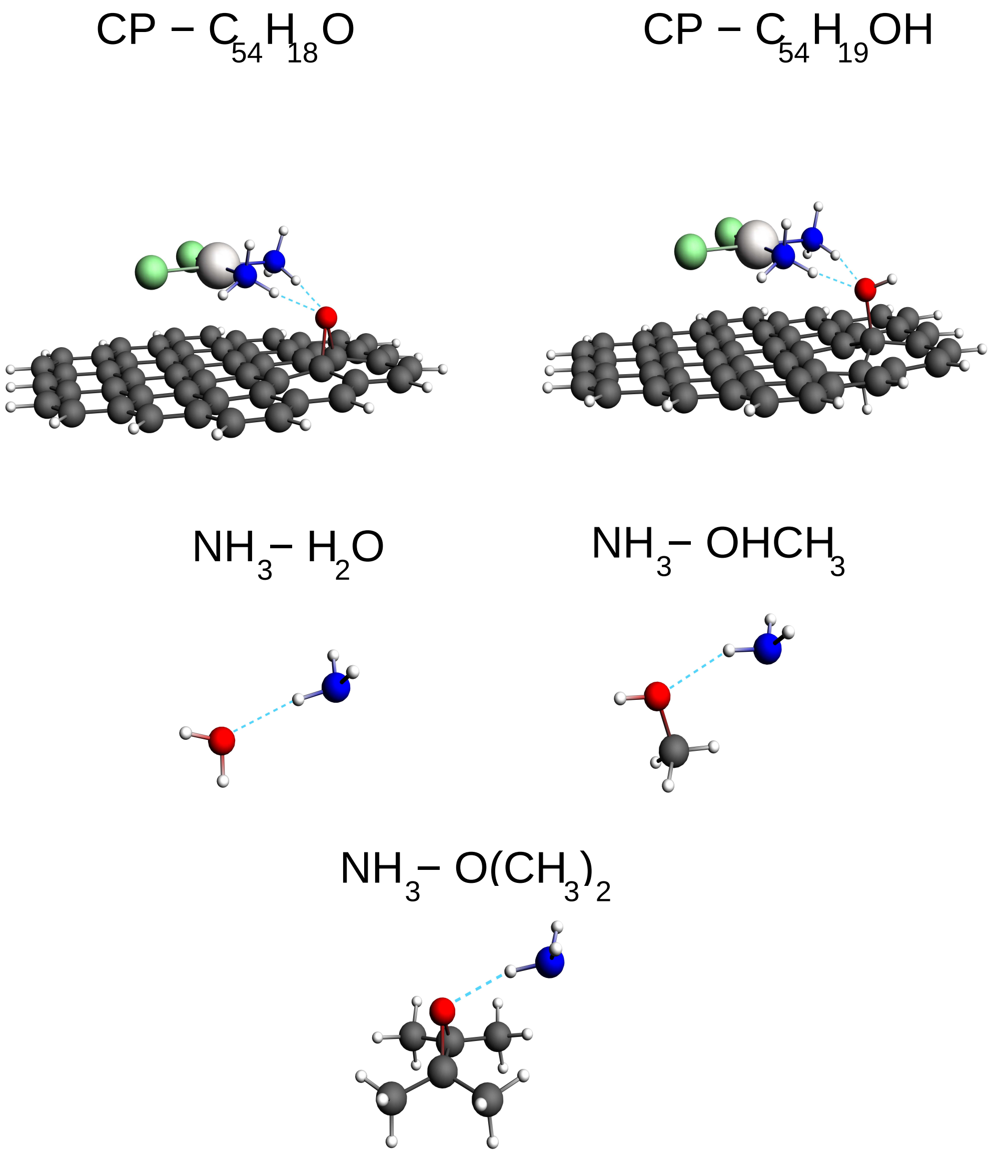

FIG. S2: Views of the optimized structures of the CP-$C_{54}H_{18}O$ and CP-$C_{54}H_{19}OH$ complexes together with those of intermolecular prototypes (NH_3-H_2O, NH_3-$OHCH_3$ and NH_3-$O(CH_3)_2$) representing archetypal hydrogen bonds. In all cases the involved hydrogen bonding is indicated by dashed lines. The partition of the total interaction energy related to the prototypical clusters involving NH_3 is reported in Table S3.
